# Changes in Pregnancy-Associated Deaths in the US During the COVID-19 Pandemic in 2020

**DOI:** 10.1001/jamanetworkopen.2022.54287

**Published:** 2023-02-01

**Authors:** Claire E. Margerison, Xueshi Wang, Alison Gemmill, Sidra Goldman-Mellor

**Affiliations:** 1 Department of Epidemiology and Biostatistics, Michigan State University, East Lansing; 2 Department of Economics, Michigan State University, East Lansing; 3 Department of Population, Family and Reproductive Health, Johns Hopkins Bloomberg School of Public Health, Baltimore, Maryland; 4 Department of Public Health, University of California, Merced

## Abstract

This cross-sectional study assesses changes in pregnancy-associated mortality from drug overdose, homicide, suicide, and other causes in the US from 2018 through 2020.

## Introduction

The COVID-19 pandemic had unique effects on pregnant and postpartum people: maternal deaths from obstetric causes increased 33% between April and December 2020 compared with previous years.^1^ That study, however, did not include deaths from nonobstetric causes among pregnant or postpartum people. Deaths from drug overdose, suicide, and homicide represent large and growing proportions of all deaths during pregnancy and the first year post partum (ie, pregnancy-associated deaths),^2^ and rates of drug overdose deaths and homicide increased substantially in the general US population in 2020.^3^ Thus, we sought to examine changes in pregnancy-associated mortality from drugs, homicide, suicide, and other causes from 2018 through 2020.

## Methods

We used cross-sectional US death certificate records from January 1, 2018, to December 31, 2020, restricted to female US resident decedents aged 15 to 44 years; deaths among women older than 44 years are more likely to be misclassified.^4^ We restricted our analysis to deaths occurring between April 1 and December 31 in each year because the COVID-19 pandemic began in March 2020. We obtained the count of live births for the same population and time frame from the Centers for Disease Control and Prevention WONDER database. This cross-sectional study followed the STROBE reporting guideline and received institutional review board approval from Michigan State University as exempt from informed consent.

The 2003 Revised Death Certificate contains a standardized pregnancy checkbox that asks whether the decedent was pregnant at the time of death, within 42 days of death, or within 43 days to 1 year of death, which we used to classify deaths as pregnancy associated. We also included deaths with *ICD-10* codes associated with death from obstetric causes (A34 and 000-099) as pregnancy associated. We classified obstetric causes, drug-related causes, suicide, homicide, and other causes using *ICD-10* codes (eTable in Supplement 1).

We calculated the pregnancy-associated death ratio as the number of pregnancy-associated deaths divided by the number of live births, multiplied by 100 000 for April to December of each year overall and for each cause of death. We generated 95% CIs assuming a χ^2^ distribution. We used StataMP, version 16 for statistical analyses.

## Results

The study included 4528 pregnancy-associated deaths. The overall pregnancy-associated death ratio from April to December 2020 was 66.9 (95% CI, 63.9-70.1) deaths per 100 000 live births, an increase of 35.0% from 2019 (Table). From 2019 to 2020, deaths from drugs increased 55.3% from 2019 to 2020; deaths from homicides, 41.2%; and deaths from obstetric and other causes (primarily motor vehicle crashes), 28.4% and 56.7%, respectively (Table). Although pregnancy-associated deaths increased over time, increases from 2019 to 2020 were substantially larger than increases from 2018 to 2019 (Table). Only pregnancy-associated suicides declined from 2019 to 2020.

**Table.** Pregnancy-Associated Death Counts and Ratios by Cause and Change Over Time From April to December in 2018, 2019, and 2020 Among US Residents Aged 15 to 44 Years

Pregnancy-associated death measure	Cause of death						Live births, No.
	Drug-related	Homicide	Suicide	Obstetric	Other	Total	
2018							
Count	205	89	82	697	227	1300	2 868 104
Ratio (95% CI)^a^	7.1 (6.2-8.2)	3.1 (2.5-3.8)	2.9 (2.3-3.5)	24.3 (22.5-26.2)	7.9 (6.9-9)	45.3 (42.9-47.9)	NA
2019							
Count	246	109	89	809	157	1410	2 843 848
Ratio (95% CI)^a^	8.7 (7.6-9.8)	3.8 (3.1-4.6)	3.1 (2.5-3.9)	28.4 (26.5-30.5)	5.5 (4.7-6.5)	49.6 (47-52.2)	NA
2020							
Count	365	147	79	992	235	1818	2 716 450
Ratio (95% CI)^a^	13.4 (12.1-14.9)	5.4 (4.6-6.4)	2.9 (2.3-3.6)	36.5 (34.3-38.9)	8.7 (7.6-9.8)	66.9 (63.9-70.1)	NA
Change, %							
2018 to 2019	21.0	23.5	9.5	17.1	−30.2	9.4	−0.8
2019 to 2020	55.3	41.2	−7.1	28.4	56.7	35.0	−4.5

Abbreviation: NA, not applicable.

^a^ Number of deaths per 100 000 live births, with 95% CI based on χ^2^ distribution.

## Discussion

We found increases in pregnancy-associated drug-related deaths and homicide and a slight decrease in pregnancy-associated suicide deaths in 2020 compared with 2018 and 2019. These findings are consistent with pandemic-related trends in the overall population and with data on obstetric deaths^1^ and pregnancy-associated homicide.^5^ These trends may reflect multiple population stressors during 2020, including COVID-19 pandemic–related economic strain, the murder of George Floyd, and the fentanyl epidemic; our analyses did not address causality. Another limitation of this study is that pregnancy-associated deaths, particularly those later in the postpartum year, were underestimated.^6^ Although pregnancy is considered an opportunity for screening and prevention related to physical, mental, and behavioral health, our data suggest that such opportunities were missed for hundreds of pregnant people during the pandemic. Our study findings suggest that there is a need for prevention and intervention efforts, including harm reduction strategies, tailored to pregnant and postpartum women, particularly during times of population stress and decreased utilization of preventive care, such as a pandemic.

## Notes

Supplement 1. **eTable.** *ICD-10* Codes Used to Define Pregnancy-Associated Deaths Due to Drug Overdose, Suicide, and Homicide

Supplement 2.
Data Sharing Statement
