# Supplementary material for: Changes in Pregnancy-Associated Deaths in the US During the COVID-19 Pandemic in 2020
Source: JAMA Netw Open. 2023 Feb 1;6(2):e2254287. doi: 10.1001/jamanetworkopen.2022.54287 (PMC9892955; doi:10.1001/jamanetworkopen.2022.54287)
Supplement: Supplement 1. — eTable. ICD-10 Codes Used to Define Pregnancy-Associated Deaths Due to Drug Overdose, Suicide, and Homicide [file jamanetwopen-e2254287-s001.pdf]

Supplementary Online Content

Margerison CE, Wang X, Gemmill A, Soldman-Mellor S. Changes in pregnancy-associated deaths in the US during the COVID-19 pandemic in 2020. *JAMA Netw Open*. 2023;6(2):e2254287. doi:10.1001/jamanetworkopen.2022.54287

eTable. *ICD-10* Codes Used to Define Pregnancy-Associated Deaths Due to Drug Overdose, Suicide, and Homicide

This supplementary material has been provided by the authors to give readers additional information about their work.

©2023 Margerison CE et al. *JAMA Network Open*.

eTable. *ICD-10* Codes Used to Define Pregnancy-Associated Deaths Due to Drug Overdose, Suicide, and Homicide

Cause	Description	Included ICD-10 codes
Drug-related death	Comprises unintentional and undetermined-intent deaths due to drug poisoning, drug-induced diseases, and mental/behavioral disorders due to drugs.[56] Drug classes include opiates & narcotics, sedatives/hypnotics, stimulants, and other psychotropics. Category *excludes* deaths coded as suicides or homicides. We chose a broad definition of drug-related deaths to account for geographic and temporal variability in coroner/medical examiner coding practices, deaths involving multiple drugs, and drug availability and lethality.	X40-X44; Y10-Y14; D52.1, D59.0, D59.2, D61.1, D64.2, E06.4, E16.0, E23.1, E24.2, E27.3, E66.1, G21.1, G24.0, G25.1, G25.4, G25.6, G44.4, G62.0, G72.0, I95.2, J70.2-J70.4, K85.3, L10.5, L27.0, L27.1, M10.2, M32.0, M80.4, M81.4, M83.5, M87.1, R50.2; R78.1-R78.5; F11.0-F11.5, F11.7-F11.9, F12.0-F12.5, F12.7-F12.9, F13.0-F13.5, F13.7-F13.9, F14.0-F14.5, F14.7-F14.9, F15.0-F15.5, F15.7-F15.9, F16.0-F16.5, F16.7-F16.9, F18.0-F18.5, F18.7-F18.9, F19.0-F19.5, F19.7-F19.9.
Suicide	Comprises all deaths with self-harm intent, including via poisoning, firearm, drowning, fire/flame, sharp/blunt object, jumping from high place, motor vehicle crash, suffocation, hanging, or other specified/unspecified means.	X60 to X84, Y87.0, or U03
Homicide	Comprises all deaths caused by another person, including via firearm, object, pushing from high place or in front of moving object, motor vehicle, neglect/abandonment, or other means. Category includes homicide via poisoning from drugs, which is historically uncommon but is increasing.	X85-Y09

©2023 Margerison CE et al. *JAMA Network Open*.
