# Supplementary material for: Changes in Pregnancy-Associated Deaths in the US During the COVID-19 Pandemic in 2020
Source: JAMA Netw Open. 2023 Feb 1;6(2):e2254287. doi: 10.1001/jamanetworkopen.2022.54287 (PMC9892955; doi:10.1001/jamanetworkopen.2022.54287)
Supplement: Supplement 2. — Data Sharing Statement [file jamanetwopen-e2254287-s002.pdf]

Data Sharing Statement

Margerison. Changes in Pregnancy-Associated Deaths in the US During the COVID-19 Pandemic in 2020. *JAMA Netw Open*. Published February 01, 2023. doi:10.1001/jamanetworkopen.2022.54287

Data
Data available: No

Additional Information
Explanation for why data not available: The data that we used is obtained with a data use agreement from the National Center for Health Statistics, and we cannot share it publicly.
